# Wisconsin Card Sorting Test: Normative data and experience

**DOI:** 10.4103/0019-5545.31582

**Published:** 2006

**Authors:** Adarsh Kohli, Manreet Kaur

**Affiliations:** * Additional Professor, Department of Psychiatry, PGIMER, Chandigarh; ** Social Scientist, Department of Psychiatry, PGIMER, Chandigarh

**Keywords:** Executive functions, WCST, norms, Indian population

## Abstract

**Background:**

The Wisconsin Card Sorting Test (WCST) has been increasingly employed as a clinical neuropsychological instrument. However, in India the use of WCST is still in a relatively preliminary stage.

**Aim:**

To analyse the utility of WCST in the Indian population.

**Methods:**

Fifty-three subjects in the age group of 20–50 years, comprising both men and women, were recruited for the study. The normality was established by administering the General Health Questionnaire as a screening instrument to evaluate their health status. The WCST was administered and the norms for various dimensions were established; these were compared with those of normal healthy individuals from the West as per the WCST manual.

**Results and conclusion:**

The significance of differences and the experience of administration have been described. The present study found highly significant differences between the means on almost all WCST scores among the Western and the Indian sample, except for the number of correct responses.

## INTRODUCTION

The Wisconsin Card Sorting Test (WCST), originally developed to assess abstract reasoning ability and the ability to shift cognitive strategies in response to changing environ-mental contingencies,^1^,^2^ is also considered a measure of the executive functions.^3^,^4^ Similar to other measures of the executive functions, WCST also requires strategic planning, organized searching, utilizing environmental feedback to shift cognitive sets, directing behaviour towards achieving a goal and modulating impulsive responding.^5^–^7^

While the WCST was developed and has been used world-wide as a measure of abstract reasoning among normal adult populations, it has also been increasingly employed as a clinical neuropsychological instrument^8^,^9^ to specifically measure brain dysfunctions affecting the frontal lobes.^10^–^14^ However, in India the use of WCST is still in a relatively preliminary stage. There is no published report of its use among Indian patients. Work is being done on patients using the WCST in the National Institute of Mental Health and Neuro Sciences (NIMHANS), Bangalore and also in the Post Graduate Institute of Medical Education and Research (PGIMER), Chandigarh. The WCST has still not been tried extensively on the Indian population. Therefore, neither its norms for India are available, nor has it been standardized or validated on the Indian population.

In a recent study the neuropsychological profile of patients with schizophrenia and normal healthy controls was assessed.^15^ Besides other tests, the WCST was administered on 20 patients with schizophrenia and 20 controls, and on comparison revealed no significant differences between the two groups. This finding led the researchers to introspect the possible reasons; for example, the chronicity and severity of illness, difficulty of administration, instructions, etc. This raises some pertinent questions regarding the WCST, such as whether it is a culture-specific test, or is it applicable to the Indian population? If yes, then is there a need for separate Indian norms?

Apart from this, one is also skeptical about the adminis-tration and scoring of the WCST. Although in the present study the standardized administration and scoring procedures as prescribed in the Wisconsin Card Sorting Manual by Heaton *et al.*^16^ were adopted, yet there were varied experiences which might prove significant in interpreting the present results.

### Aim

To analyse the utility of WCST among the Indian population.

### Objectives

1. To share the experience of administration and scoring of WCST on the Indian population.
2. To formulate normative data on WCST for use in India.

## METHODS

### Sample

Fifty-three healthy subjects were recruited for administration of the WCST. These healthy subjects were administered the General Health Questionnaire^17^ for evaluating their health status.

### Design

Cross-sectional, assessment

### Instruments

1. Wisconsin Card Sorting Test^16^
  The WCST is a neuropsychological instrument used to measure the executive functions, reportedly sensitive to brain dysfunction affecting the frontal lobes.
2. General Health Questionnaire-12^17^
  It is a screening instrument to evaluate the health status of individuals. A cut-off score of 2 or above implies pathology.

### Procedure

Normal healthy subjects were contacted and the purpose of the study was explained to them. Written informed consent was obtained and confidentiality ensured. The General Health Questionnaire^17^ was administered to them for evaluating their health status and the WCST was administered. It took about 30-45 minutes to complete the test.

## RESULTS

### Statistical Analysis

Basic statistics in the form of mean and SD were computed for all variables; *t*-test was computed to ascertain differences on WCST scores between the two groups.

### Description of the sample

Fifty-three normal subjects (27 men, 26 women) were recruited. Their mean age was 32.98 years (SD 8.69) and mean education was 13.69 years (SD 2.60) (Tables 1 and 2).

**Table 1:** The Wisconsin Card Sorting Test: Sample characteristics of men for age and education category

	Men											
	
	20–30 years				31–40 years				41–50 years				
			
	School educated		College educated		School educated		College educated		School educated		College educated	
						
*n*	9		3		3		6		3		3	
						
	Age	Education	Age	Education	Age	Education	Age	Education	Age	Education	Age	Education
Mean	24.22	11.00	23.67	15.33	35	10	33.83	16.17	44.5	10	45.67	16	
SD	3.07	1.00	3.06	0.58	1.41	0.00	3.31	1.47	0.71	0	2.51	1.73

**Table 2:** The Wisconsin Card Sorting Test: Sample characteristics of women for age and education category

	Women											
	
	20–30 years				31–40 years				41–50 years			
			
	School educated		College educated		School educated		College educated		School educated		College educated	
						
*n*	4		6		3		5		3		5	
						
	Age	Education	Age	Education	Age	Education	Age	Education	Age	Education	Age	Education
Mean	24.22	11.33	24.66	14.83	36	11	34.6	15.2	42	10	45	15.6
SD	3.05	0.58	3.01	1.60	1.0	1.0	3.36	0.45	0.58	0	4.85	0.89

To formulate normative data for WCST among the Indian population, the scores of the 53 subjects were compared with a Western group of 63 subjects, with a comparable mean age of 34.46 years (SD 2.68) and mean education of 15.68 years (SD 3.03). Results in Table 3 reveal highly significant differences between the mean values of the two groups.

**Table 3:** Mean, SD and *t*-values of scores on the Wisconsin Card Sorting Test of Indian and Western subjects

S. No.	Variables	Indian *n*=53	Western *n*=63	*t*-value
1.	No. of categories completed	M=4.29	M=5.62	5.32**
		SD=1.62	SD=1.08	
2.	Total no. of trials administered	M=114.25	M=84.81	8.56**
		SD=18.05	SD=18.98	
3.	Correct response	M=71.22	M=68.65	1.11
		SD=13.88	SD=10.58	
4.	Errors	M=42.96	M=16.16	7.81**
		SD=21.74	SD=13.31	
5.	% Errors	M=35.66	M=17.57	7.32**
		SD=15.09	SD=10.74	
6.	Perseverative response	M=28.05	M=8.87	6.95**
		SD=18.57	SD=8.28	
7.	% Perseverative responses	M=23.06	M=9.40	6.76**
		SD=13.69	SD=5.84	
8.	Perseverative errors	M=24.49	M=8.29	7.33**
		SD=14.78	SD=7.00	
9.	% Perseverative errors	M=20.17	M=8.89	6.96**
		SD=10.92	SD=4.79	
10.	Non-perseverative errors	M=18.16	M=7.87	5.78**
		SD=11.02	SD=7.43	
11.	% Non-perseverative errors	M=15.74	M=8.68	4.55**
		SD=8.71	SD=7.77	
12.	Trials to complete 1st category	M=17.17	M=12.17	2.09*
		SD=16.85	SD=4.76	
13.	Failure to maintain set	M=1.19	M=0.57	2.82**
		SD=1.29	SD=1.10	
14.	Learning to learn	M=-5.35	M=-1.46	4.14**
		SD=6.15	SD=3.35	
15.	Conceptual level responses	M=57.5	M=78.76	7.43**
		SD=16.13	SD=14.34	

* p<0.05;

** p<0.01

## DISCUSSION

The WCST, a test of the executive functions, is new in the history of neuropsychological testing in India but with limited use. Therefore, a sample was taken to relate the experience encountered in administering and scoring of WCST and to establish normative parameters for Indian men and women.

The procedure followed for the administration of WCST was in accordance with the directions given in the WCST manual. Since the population belonged to different cultural backgrounds, instructions in the present study were given to the subjects in English, Hindi as well as Punjabi—a direct translation from the version in English by Heaton *et al.*^16^

During the administration of WCST it was experienced that the clients exhibited a kind of lack of understanding of initial conceptualization of WCST, akin to those from the patients' sample.^15^ Instead of matching with the stimulus cards at times they just randomly dealt with the response cards and the examiner had to stop the client, insisting him to look at the stimulus cards and try to match them. Moreover, the clients sometimes became perplexed about how to form response card piles below the stimulus cards. They tended to either form columns below the stimulus cards, or began to stack the response cards on top of the stimulus cards. They also tended to match the new response cards to the top card piles, rather than the stimulus cards, leading to ‘other’ responses. It was also observed that a few of them could not complete a single set, while the first two principles/sets (colour, form) were relatively easily learnt by most of them, whereas the ‘number’ principle was learnt with relatively more difficulty. Also, in case of three completed sets, the fourth set (of colour again) was all the more difficult to achieve as they made more errors and took more trials. Here the subjects seemed confused and apparently looked for another fourth principle in sorting (after colour, form and number). There was a cognitive explanation in the form of ‘expectancy’ or prediction of something more forthcoming.

Interestingly, it was also observed that although the first set was learnt in relatively lesser number of trials, yet the percentage of errors increased with successive trials. And, those who caught on the trick were found to generally finish with just 1–2 errors per set. It was like either you get the ‘hang of it’ or it showed some kind of a mental block.

The other aim of the study was to formulate the normative data for WCST. Comparing the scores of normal Indian subjects with comparable Western normal subjects was another interesting cross-cultural aspect of the study. The present study reports highly significant differences between the mean values on almost all WCST scores among the Western and Indian samples, except for the number of correct responses. The subjects of the Western sample were screened for history of neurological dysfunction, learning disability or other chronic organic conditions. The sample is the normative sample from the original WCST manual.^16^ The Indian subjects not only completed lesser number of categories, but also made more errors, took more trials and were more perseverative. As compared to their western counterparts, as they had lower conceptual level response score, and problems in maintaining a consistent problem-solving strategy within categories (failure to maintain set) and they were also less efficient with sorting as the test progressed.

Although a successful performance on the WCST requires a complex set of cognitive functions, including the ability to think abstractly, selectively attend to a particular perceptual dimension and a shift in cognitive set. There is early evidence about the relationship of WCST and everyday life impairments.^18^ Yet, because of lack of relevant Indian data the above results can in no way be interpreted as some kind of cognitive dysfunction among the Indian sample. Rather, the question arises about the culture-free status of the WCST, raising doubts about its direct application to the Indian population; maybe there is a need for adaptation. This change can be in terms of modified instructions, construction of valid norms, relevant to Indian subjects. It does indicate a need for more research in the area of WCST and its utility in the neuro-psychological assessment and rehabilitation.

In one study, Spanish-speaking participants from the US–Mexico borderland and Spain were compared on neuro-psychological measures. Results indicated that living in the USA was positively correlated with performance on WCST. The WCST measures abilities that are emphasized more in US schools than in Mexican schools. Mexican schools emphasize rote memorization and focus less on problem-solving than do schools in the USA.^19^ Similarly, lower scores of the Indian population on WCST can also be attributed to more emphasis on rote memorization in India, which avoids grasping the inner complexities and inferences of the subject that is being learned and instead focuses on memorizing the material for later recall.

## CONCLUSION

The effectiveness of WCST is doubtful in the present rapidly changing environment. Traditionally, schools in India are examination-oriented; they provide little scope for personality development, creative abilities and cultivation of airs. Further research is warranted with a larger sample to prove the efficacy of WCST and its use as a neuropsychological tool and in rehabilitation.
